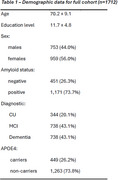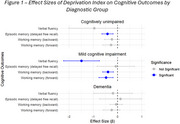# Associations of neighborhood disadvantage in Barcelona with memory and executive functions in cognitively unimpaired older adults and patients with mild cognitive impairment and dementia

**DOI:** 10.1002/alz70860_102936

**Published:** 2025-12-23

**Authors:** Carmen M Colceriu, Pablo Aguilar, Alex López, Eleni Palpatzis, Judith Garcia‐Aymerich, Mark Nieuwenhuijsen, Adrià Tort‐Merino, María Franquesa‐Mullerat, Sara E Zsadanyi, Antonia Valentín, Sami Petricola, Juan Fortea, Alberto Lleó, Neus Falgàs Martínez, Raquel Sánchez‐Valle, Alexandre Bejanin, Eider M Arenaza‐Urquijo

**Affiliations:** ^1^ Global Health Institute Barcelona (ISGlobal), Barcelona, Spain; ^2^ University of Pompeu Fabra (UPF), Barcelona, Spain; ^3^ Sant Pau Memory Unit, Department of Neurology, Hospital de la Santa Creu i Sant Pau, Institut d'Investigació Biomèdica Sant Pau (IIB SANT PAU), Facultad de Medicina ‐ Universitat Autònoma de Barcelona, Barcelona, Spain; ^4^ Alzheimer's Disease and Other Cognitive Disorders Unit, Neurology Department, Hospital Clinic, Barcelona, Spain; ^5^ Sant Pau Memory Unit, Hospital de la Santa Creu i Sant Pau, Institut de Recerca Sant Pau ‐ Universitat Autònoma de Barcelona, Barcelona, Spain; ^6^ Centro de Investigación Biomédica en Red de Fragilidad y Envejecimiento Saludable (CIBERFES), Madrid, Spain

## Abstract

**Background:**

Neighborhood disadvantage is associated with heightened dementia risk and worst cognitive outcomes. We evaluated associations between deprivation index (DI) ‐ reflecting neighborhood disadvantage ‐ and cognition, and explored whether these are driven by amyloid pathology in cognitively unimpaired (CU) older adults and patients with mild cognitive impairment (MCI) and dementia.

**Method:**

We included 1712 participants from UBRAIN study utilizing data from Clinic and Sant Pau Hospitals in Barcelona ‐ 20.1% CU, 43.1% MCI, 36.8% dementia patients (54% Alzheimer's Disease, 24% Frontotemporal, 12% Lewy body, other 10%) (Table 1). Episodic memory (FCSRT delayed free recall), working memory (WAIS Digits forward and backward) and verbal fluency were considered as outcomes. DI was calculated based on a validated measure using 6 indicators (e.g. employment, internet access). We ran linear regression models, both in the full sample and in CU, MCI and dementia patients separately, adjusted by sex and age and assessed the effect education and CSF‐derived amyloid status ‐ positive (Aβ+) or negative (Aβ‐).

**Result:**

In the full sample, higher DI was associated with lower performance in verbal fluency (β=‐0.858, *p* <0.01), WAIS‐Digits forward and backward (β_1_=‐0.286, p_1_<0.01; β_2_=‐0.206, p_2_<0.01), independent of age and sex, but, the associations were accounted for by educational level (p_1_=0.35, p_2_=0.1, p_3_=0.29). When stratifying by diagnostic group, however, the associations remained independent of education. In CU, we observed a significant association between ADI and FCSRT delayed free recall (β=‐0.306, *p* = 0.011) and, in MCI, with verbal fluency (β=‐1.075, *p* = 0.01) and WAIS‐Digits backward and forward (β_1_=‐0.384, p_1_=0.03; β_2_=‐0.29, p_2_=0.036, Figure 1). Associations in CU were driven by Aβ‐ participants (*p* = 0.036) and in MCI by Aβ+ participants (*p* = 0.04).

**Conclusion:**

Older adults living in more socially disadvantaged areas in Barcelona might be at higher risk of future cognitive decline. Further, the effect of social disparities might be more pronounced before dementia onset in CU and MCI participants.